# Clinical Use of Epidermal Growth Factor Receptor Testing in Patients With Advanced Lung Cancer by Physicians: Survey of US and International Patterns

**DOI:** 10.1200/JGO.18.00057

**Published:** 2019-02-27

**Authors:** Matthew Peters, Edward S. Kim, Vera Hirsch

**Affiliations:** ^1^ Macquarie University, Sydney, New South Wales, Australia; ^2^ Levine Cancer Institute, Carolinas HealthCare System, Charlotte, NC; ^3^ McGill University Health Centre, Montreal, Quebec, Canada

## Abstract

**PURPOSE:**

Guidelines recommend testing for *EGFR* mutation at diagnosis of advanced non–small-cell lung cancer to guide treatment. Two surveys, 18 months apart, aimed to identify changes in *EGFR* mutation testing and treatment practices in non–small-cell lung cancer.

**METHODS:**

The first survey of 562 physicians from Canada, France, Germany, Italy, Japan, South Korea, Spain, Taiwan, the United Kingdom, and the United States was conducted between December 2014 and January 2015. The second, between July and August 2016, surveyed 707 physicians in the same countries with the addition of China; China was excluded from year-on-year comparisons.

**RESULTS:**

Globally (excluding China), physicians requested *EGFR* mutation testing in 80% (excluding China; 2015: 81%) of patients before first-line therapy. In 2016, 18% of results were not received before initiating treatment, a significant improvement over 2015 (23%). Reasons for not testing included tumor histology, insufficient tissue, poor performance status, and long turnaround time, although this had significantly improved in 2016 from 2015. Prolonging of survival/extending life was deemed the most important therapy goal in first-line treatment of both cohorts.

**CONCLUSION:**

Improvements in availability of test results before first-line therapy were seen, but incomplete implementation of guidelines is still observed, resulting in a large proportion of patients not receiving tyrosine kinase inhibitor treatment on the basis of mutation status. The reasons for not testing remained the same, year-on-year: tumor histology, insufficient tissue, poor performance status, and long test turnaround time. Receiving timely results must be addressed, if treatment parity for eligible patients can be achieved. Physician education and closer guideline concordance are key steps to improve outcomes.

## INTRODUCTION

Lung cancer is the leading cause of cancer-related mortality, accounting for approximately 1.59 million deaths per year worldwide, with most patients dying within 12 months of diagnosis.^1^ Improving survival for the majority of patients who have advanced disease at the time of diagnosis requires a deep understanding of lung cancer biology and the development of novel effective treatments that can be matched to a specific tumor characteristic with a readily available diagnostic test. The potential benefit of treatment can be maximized only if there are the highest standards of diagnostic practice and the consistent application of optimal treatment on the basis of defined tumor biology.

At present, one of the most important biomarkers is the presence of specific genetic alterations in the *EGFR* gene that confer treatment sensitivity to epidermal growth factor receptor (EGFR)–tyrosine kinase inhibitors (TKIs).^2,3^ The prevalence of *EGFR* mutations in non–small-cell lung (NSCLC) tumors varies according to ethnicity: in white patients it ranges from 10% to 15%^4,5^; in African American patients, 19%^6^; in Asian populations, 40% to 50%.^7-10^ The most clinically significant *EGFR* mutations are either deletions in exon 19 (del19) or the L858R substitution mutation (together they represent 80% to 90% of *EGFR* mutations).^3^

Clinical trial results evaluating treatment with EGFR-TKIs highlight the importance of patient selection for novel treatments. In unselected patients with advanced NSCLC, the TKIs gefitinib and erlotinib produced response rates of 8% to 9%, with a median time to progression of 2.2 months to 3.0 months.^11^ In contrast, in *EGFR* mutation–positive patients, response rates of 68%, mean progression-free survival (PFS), and time to progression of 12 months were observed in patients treated with gefitinib and erlotinib.^11^ Proving EGFR-TKIs improve overall survival has been challenging, but in trials in patients with metastatic disease whose tumors have activating *EGFR* mutations, high response rates (approximately 70%) and significantly longer PFS have been seen in patients treated with EGFR TKIs (gefitinib, erlotinib, afatinib) as first-line treatment when compared with those receiving chemotherapy.^3^ These kinds of results have helped establish the use of EGFR-TKIs in clinical practice, and routine testing of appropriate cases for *EGFR* mutations is recommended by international guidelines from the College of American Pathologists, the International Association for the Study of Lung Cancer, the Association for Molecular Pathology,^11^ and the European Society for Medical Oncology.^12^

The diagnostic work-up in patients with breast cancer routinely includes hormone status and *HER2-neu*.^13^ Many experts would argue that making a treatment decision without this information would not be ethical. However, pretreatment testing is not always undertaken in patients with lung cancer. A retrospective survey of records from patients with NSCLC tested for *EGFR* mutations during 2011 in 11 Asian Pacific countries found that only 31.8% of patients were tested.^14^ A Swedish study reviewing data from 2010 to 2012 found only 49% of patients with advanced-stage NSCLC with nonsquamous histology were referred for EGFR analysis, despite national guidelines recommending EGFR analysis.^15^ Because EGFR testing has become more prevalent, we sought to poll physicians to assess the current landscape.

In 2015, an international survey concluded that, despite guidelines, not all patients with NSCLC were tested and received test results before treatment initiation, with country and regional variances in testing rates.^16^ To identify year-on-year changes, an additional international survey was conducted in 2016.

## METHODS

### Country Scope

To assess both global trends and regional differences regarding *EGFR* mutation testing, we administered two surveys in 2015 and then in 2016. Both surveys were multicountry, including North America (United States, Canada), Western Europe (France, Germany, Italy, Spain, United Kingdom), and Asia (Japan, South Korea, Taiwan); China was added in the 2016 survey. The countries were chosen to ensure a range of ethnicities were included and to focus on countries with an established infrastructure of *EGFR* mutation testing.

### Data Collection

Data were collected through a structured online self-reporting questionnaire addressed to oncologists, pulmonologists, and thoracic/respiratory surgeons. Fieldwork took place between December 16, 2014 and January 16, 2015 and between July 15 and August 19, 2016. Physicians qualified if they had treated a minimum number of patients with stage IIIb to IV NSCLC in the preceding 3 months (see Appendix for additional detail). All participants personally decided the treatment of patients with NSCLC or were actively involved in the decision. All interviews were conducted online. To avoid any bias in results, the invitation text referred to treatment of advanced NSCLC and did not include any information about *EGFR* mutation testing.

### Contents of Questionnaire

The questionnaire addressed five themes.

- Number of patients with stage IIIb/IV *EGFR* mutation–positive disease allocated to therapy lines and treatment regimens used in first-line and later lines of therapy.
- Percentage of patients with first-line stage IIIb/IV NSCLC for whom an *EGFR* mutation test was ordered before first-line therapy.
- *EGFR* mutation subtypes considered by physicians, including the answer category “I do not consider this level of detail.” Physicians who considered mutation subtypes were asked in a yes-no question whether the subtype affected their treatment decision.
- Importance of different features of treatment regimens on therapy choice in first-line therapy of *EGFR* mutation–positive patients.
- Therapy goals in first-line therapy of *EGFR* mutation–positive patients.

### Statistical Analysis

Descriptive statistics were used to summarize the responses to each question on global and regional levels. Subgroup comparisons (differences between regions) were analyzed with χ^2^ tests and *t* tests. To reach global and regional totals and representative averages regarding the size of the included countries, respondent data were weighted by the country using a commercially sourced summary of incidence of NSCLC.^17^ In addition, within-country weightings were applied by hospital types (eg, number of academic hospitals *v* community hospitals in a given country), specialty (eg, oncologists *v* pulmonologists in France and Germany), and practice setting (eg, private practice *v* hospital in Germany). Because China was a newly added country, the majority of results track year-on-year comparisons and, therefore, exclude China for comparison purposes.

## RESULTS

### Sample Description

Across all countries (10 in 2015, 11 in 2016), physicians (18,831 in 2015, 11,421 in 2016) were contacted through one global panel provider covering all countries involved and being supported by additional local providers for the United Kingdom, Canada, Japan, South Korea, Taiwan, and China. Of those contacted, 1,166 (6.2%) and 1,359 (11.9%) physicians agreed to participate in 2015 and 2016, respectively; 604 (2015) and 652 (2016) did not meet the screening criteria or did not complete the survey, leaving 562 (2015) and 707 (2016) physicians in the analysis (2015; North America: n = 161; Europe: n = 251; Asia: n = 150, 2016; North America: n = 162; Europe: n = 303; Asia: n = 242). The respondents were 73% (2015) and 71% (2016) oncologists, 25% (2015) and 22% (2016) pulmonologists (from France, Germany, Japan, South Korea, and Taiwan), 2% (2015) and 4% (2016) thoracic/respiratory surgeons (Japan and China), and 3% (2016) respiratory specialists (China). The respondents reported managing, in total, 88,756 patients with stage IIIb/IV NSCLC in the preceding 3 months (2015: 33,327; 2016: 55,429), an average of 70 patients per physician (2015: 59 patients; 2016: 78 patients).

### *EGFR* Mutation Testing

The number of tests ordered but results not received before first-line treatment improved year-on-year, dropping down significantly from 23% in 2015 to 18% in 2016 (*P* < .01). In the 2016 survey, respondents were asked how quickly results were available: for the majority of patients tested for *EGFR* mutations, results were available within 10 business days, but 24% (excluding China) of test results were received later. This share was significantly lower in Asia (excluding China; 16%) than in Europe (32%; *P* < .01) and in North America (24%; *P* < .05).

Overall, little had changed in terms of *EGFR* mutation testing: in 2016, testing before first-line therapy was requested in 80% (excluding China) of patients; in 2015 that figure was 81% of patients with stage IIIb/IV NSCLC.^18^ Testing frequency differed between regions. In Asia, an *EGFR* mutation test was ordered before first-line therapy in 84% of patients in 2016, a significant drop from 92% in 2015 (*P* < .01). In Europe, this happened in 81% of patients in 2016, not significantly changed from 77% in 2015. In North America, the figure barely changed year-on-year (2016: 77% *v* 2015: 76%).

There are a number of reasons not all patients with advanced NSCLC were tested before first-line therapy. Besides histology, in squamous cell carcinoma for example (2016: reported by 58% of the physicians *v* 2015: 70%), the main barriers were insufficient tissue (2016: 63% *v* 2015: 66%), poor performance status (2016: 40% *v* 2015: 37%), and long turnaround time (2016: 21% *v* 2015: 30%). In addition, 21% of the physicians not testing all their patients do not test because they believe the results would not have an impact on the therapy decision (Asia [excluding China] 10%; Europe 20%; North America 26%). The 2016 survey showed there had been an improvement in mutation test results available before treatment, increasing to 82% from 77% of the patients for whom a test was ordered before first-line treatment.

### Impact of *EGFR* Mutation Test Results on Treatment Decisions

Globally (excluding China) in 2016, 79% (2015: 80%) of *EGFR* mutation–positive patients were treated first line with TKIs with regional variances: North America 2016: 73% (2015: 83%); Europe 2016: 77% (2015: 76%); Asia 2016 (excluding China): 88% (2015: 81%). In China, however, only 45% of *EGFR* mutation–positive patients received TKIs in first-line treatment.

The number of physicians who agreed that the mutation subtype influenced treatment decisions had increased globally (excluding China) from 49% in 2015 up to 60% in 2016 (*P* < .01), driven largely by notably changing attitudes in Europe, where 58% of respondents agreed with this view in 2016, up from just 40% in 2015 (*P* < .01). The majority of physicians in Asia (excluding China) held this view (2016: 79%; 2015: 72%); 67% of physicians in China agreed that subtype influenced treatment decisions, whereas only 47% of physicians in North America did, although this was not a substantial increase from 40% in 2015.

### Criteria of Treatment Choice in First-Line Therapy

The outcome of *EGFR* mutation testing was only one of many factors informing treatment decisions. Patient characteristics (eg, performance status, speed of tumor progression, expected compliance), perceived strengths or weaknesses of available treatment regimens from different sources of information, and, finally, therapy goals are common criteria and influencing factors for treatment decisions in individual patients.^19^

The majority of physicians agreed that prolonging survival/extending life was the most important goal in first-line therapy of advanced NSCLC. In line with this, an increase in overall survival was by far the most important criterion in physician’s choice of a first-line treatment of *EGFR* mutation–positive patients (2016: 54% *v* 2015: 49%). Other factors cited as the most important criterion of treatment choice were an increase in PFS (2016 [excluding China] and 2015: 18%) and strong improvement of health-related quality of life (2016 [excluding China] and 2015: 8%). In the 2016 survey, 51% of respondents did not differentiate between available *EGFR*-targeted therapies; however, data are now available to help physicians make informed treatment decisions between first- and second-generation TKIs.^20^

## DISCUSSION

Inevitably, there is a transition phase when new data establish an updated practice paradigm for the investigation and management of any condition. The rate of transition should be rapid when outcomes are clearly beneficial on the basis of optimal clinical care. Although the two surveys do show a year-on-year improvement, some patients with advanced lung cancer carrying activating *EGFR* mutations appear to receive care that is not in line with current evidence and guidelines.

Surveys such as this do have limitations. Taiwan, Korea, and Japan have well-resourced health care systems, and the findings from these countries should not be taken as representative of Asia more generally, given the cultural and economic diversity. However, year-to-year comparisons are valid for these countries, and they provide a basis for comparison with Europe/North America. We had a lower cutoff for patients with lung cancer per physician. We believe that the first educational task is to ensure that physicians who frequently see patients with lung cancer are practicing optimally. Local health care systems should have rules and standards to protect patients from substandard care from physicians who only occasionally manage lung cancer. Physicians practicing in academic rather than community settings may have different knowledge and behaviors. This is a question for future research; we could not scale this survey to answer this important question.

In many aspects, the situation in the Asian countries studied, with the exception of China, differs from that in Europe and North America. Possible explanations for this include higher detection rates for *EGFR* mutations, services for molecular diagnosis that are more efficient, more physician experience with first-line EGFR-TKI treatment, increased observation of clinical benefits, and more experience with the management of adverse effects in Asia. Many clinical trials demonstrating superiority of EGFR-TKIs over cytotoxic chemotherapy were conducted in Asia, and it may also be the case in Asia that patient expectations interact with those of physicians so as to increase the likelihood of testing and treating patients in accordance with guidelines.

It is disappointing that adequacy of sample remains a significant barrier to test performance. Optimal tissue collection for marker assessment, plus histologic assessment, is paramount to diagnosing and staging patients with lung cancer. Developments may occur in the area of blood testing for circulating tumor DNA. This may be an advantage if it reduces time between clinical assessment and the molecular genetic testing result and yields an accurate test outcome.

In both surveys, respondents cited histology as one of the reasons not to test for *EGFR* mutations. Neither survey examined the reasoning for not testing before first-line treatment, but the assumption is that the lower frequency of detection in squamous cell carcinoma contributed to this practice. *EGFR* mutations are detected in some squamous cell carcinomas.^21^ However, when the rate of a positive test outcome is low, the health economics around testing are altered, and respondents in some countries cited cost as a reason patients were not tested. Furthermore, EGFR testing is currently only recommended in certain subgroups of squamous NSCLC, such as nonsmokers,^3^ and in selected countries histology may affect reimbursement for testing.

The need for some or all patients with NSCLC to have molecular tumor analysis can extend the period between execution of a biopsy and the delivery of all clinical knowledge on which a treatment choice should be made. More Asian physicians waited until results were available, and only 16% (2016, Asia [excluding China]) of the test results were delayed beyond 10 business days. When treatment-defining mutations are more common, diagnostic services can be organized to deliver results rapidly. Part of the modern physician’s clinical skill set must be to explain to patients the need for this delay, the comparative benefit of optimal treatment, and the small or absent harm from delaying treatment a short time, if those physicians who initiate treatment before results are available do so because of patient pressure. However, laboratory services need to be efficient in both testing and communication of results. A small proportion of respondents in all regions reported that they did not test all their patients before first-line therapy for *EGFR* mutation because they believed the results would not have an impact on therapy decision (2016; globally [excluding China] reported by 12% of the physicians, 16% North America, 13% Europe, and 5% Asia [excluding China]). The survey results do not provide the reasons behind this thinking, but it is simple nonadherence to guidelines and not a failure of the laboratory service. Educational efforts are desperately needed for this group.

When patients tested positively for an *EGFR* mutation, there was a variable use of mutation status to inform treatment decision. Large numbers of European and US physicians reported that this level of detail was of no interest to them, but this was rarely the case in Asia. It is possible that some physicians are slow to change prescribing habits or are making a trade-off on the basis of their perceptions of treatment tolerability. If it is the latter, it does not track their professed treatment aim, which, for the majority of respondents in both surveys, was prolonging survival and/or extending life. From the patient’s perspective, it is important to ensure that any real or perceived survival/tolerability trade-off is made only after detailed discussion.

Lung cancer treatment, as with many other cancers, has become more precise, using molecular genomic results to determine optimal therapy—whether at this time it is targeted therapy or cytotoxic chemotherapy. This has been occurring in a number of cancers, including chronic myeloid leukemia, breast, colon, and melanoma. We observe areas of clinical practice that are suboptimal, even though only simple changes are needed to achieve concordance with guidelines. This should be a concern at a time of rapid change in the knowledge of lung cancer biology. Novel resistance mechanisms, second- and third-line treatment options, and the need for repeated biopsies, together with the potential for molecular testing from blood samples, are knowledge and practice changes that would not have been contemplated a decade ago. They offer the prospect of much-improved clinical outcomes for patients with lung cancer. From the patient perspective, they want their caring clinicians to be at the forefront of practice—not to be a slow adopter. Gaps between the generation of knowledge, its dissemination to clinicians, and changes in the clinical care that they deliver must be closed.

## Data Availability

The following represents disclosure information provided by authors of this manuscript. All relationships are considered compensated. Relationships are self-held unless noted. I = Immediate Family Member, Inst = My Institution. Relationships may not relate to the subject matter of this manuscript. For more information about ASCO's conflict of interest policy, please refer to www.asco.org/rwc or ascopubs.org/jco/site/ifc. **Consulting or Advisory Role:** GlaxoSmithKline **Speakers' Bureau:** AstraZeneca **Expert Testimony:** GlaxoSmithKline **Honoraria:** Celgene, Eli Lilly, AstraZeneca, Boehringer Ingelheim, Pfizer, Merck **Consulting or Advisory Role:** Eli Lilly, Celgene, AstraZeneca, Boehringer Ingelheim, Pfizer, Merck **Research Funding:** Boehringer Ingelheim, Eli Lilly, Merck, Ignyta, Genentech **Travel, Accommodations, Expenses:** Eli Lilly, Celgene, AstraZeneca, Boehringer Ingelheim No other potential conflicts of interest were reported.

## Appendix

### Eligibility Criteria for Participation in the Survey

Physicians were asked how many unique adult patients with advanced/metastatic (stage IIIb/IV independent of histology and treatment line) non–small-cell lung cancer (NSCLC) they had actively managed (including those who may be receiving best supportive care/palliative treatment) in the last 3 months. If they met the following criteria, they qualified to participate in the survey:

- United States, France, United Kingdom: the number of patients with advanced NSCLC was 10 or more
- Italy: the number of patients with advanced NSCLC was 15 or more
- South Korea: the number of patients with advanced NSCLC was 40 or more
- Spain, Japan, Canada, Taiwan: the number of patients with advanced NSCLC was seven or more
- Germany:
  - If Hospital Oncologist (S2 code 1 or 5 AND S5 code 15-19): the number of patients with advanced NSCLC was 30 or more
  - If pulmonologists (S2 Code 7) OR office-based oncologist (S2 code 1 or 5 AND S5 code 20-23) the number of patients with advanced NSCLC was 20 or more
- China: the number of patients with advanced NSCLC was nine or more

The respondents reported managing, in total, 88,756 patients with stage IIIb/IV NSCLC in the preceding 3 months (2015: 33,327; 2016: 55429), an average of 70 patients per physician (2015: 59 patients; 2016: 78 patients). Of these patients, 5,106 (2015) and 10,605 (2016) were *EGFR* mutation positive, which represents globally 15.3% (2015) and 19.1% (2016) of patients with NSCLC in advanced stages (2015; North America: 13.4%; Europe: 13.1%; Asia: 24.2%, 2016; North America: 18.5%; Europe: 16.3%; Asia: 24.8%).
